# Factors Associated with Post-Progression Survival in Patients with Advanced Hepatocellular Carcinoma Treated with Sorafenib

**DOI:** 10.3390/diseases3020068

**Published:** 2015-05-15

**Authors:** Taiga Otsuka, Shunya Nakashita, Kimihiko Yanagita, Keisuke Ario, Hiroaki Kawasoe, Seiji Kawazoe, Yuichiro Eguchi, Toshihiko Mizuta, the Saga Liver Cancer Study Group

**Affiliations:** 1 Department of Internal Medicine, Hepatology Division, Saga University Hospital, 5-1-1 Nabeshima, Saga 849-8501, Japan; E-Mails: shunya0625@mac.com (S.N.); mizuta1221@gmail.com (T.M.); 2 Department of Internal Medicine, Saiseikai Karatsu Hospital, 817 Motohata-machi, Karatsu 847-0852, Japan; E-Mail: k_yanagt@po.saganet.ne.jp; 3 Department of Internal Medicine, Gastroenterology Division, NHO Ureshino Medical Center, 2436 Shimojuku-hei Ureshino-machi, Ureshino 843-0393, Japan; E-Mail: ariok@ureshino.go.jp; 4 Department of Internal Medicine, Karatsu Red Cross Hospital, 1-5-1 Futago, Karatsu 847-8588, Japan; E-Mail: kawazoe-hiroaki@imari-arita-hp.or.jp; 5 Department of Hepatobiliary and Pancreatology, Saga-Ken Medical Centre Koseikan, 400 Nakabaru, Kase-machi, Saga 840-8571, Japan; E-Mail: kawazoe-s@excite.co.jp; 6 Liver center, Saga University, 5-1-1 Nabeshima, Saga 849-8501, Japan; E-Mail: eguchiyu@me.com

**Keywords:** beyond progression, progressive disease, scoring system, post-progression survival

## Abstract

Sorafenib exerts modest antitumor activity in patients with advanced hepatocellular carcinoma (HCC), and radiological progressive disease (rPD) does not always correspond to so-called clinical progressive disease (cPD). We evaluated 101 patients who initiated sorafenib treatment for HCC and assessed post-progression survival (PPS) using the Cox proportional hazards model. PPS was calculated from the date of the first rPD until the date of death or the last follow-up. Using Cox model analysis of the 76 patients who experienced first rPD, we identified the Child-Pugh class, Eastern Cooperative Oncology Group performance status, the best antitumor response during treatment (using Response Evaluation Criteria in Solid Tumors (RECIST) Version 1.1) and α-fetoprotein levels as independent factors affecting PPS. When these factors were used to define scores ranging from zero to five with a cutoff value of two, PPS of patients who received best supportive care (BSC) after rPD was not statistically significantly different from that of patients who received post-rPD therapy with scores ≥2 (*p* = 0.220). In contrast, the PPS for the post-rPD therapy group was significantly longer compared with the BSC patients with scores <2 (*p* < 0.001). Patients who scored ≥2 at their first rPD were judged cPD and as candidates for BSC.

## 1. Introduction

Hepatocellular carcinoma (HCC) is one of the most common cancers worldwide [1,2]. The prognosis of HCC depends on its stage at diagnosis. Although the prognosis is favorable for patients with early HCC who receive radical therapy, it is poor for those with advanced HCC. Sorafenib is the first targeted agent with significant clinical activity for advanced HCC. In previous multicenter, double-blind, randomized phase 3 studies, the SHARP [3] and Asia-Pacific [4] studies, sorafenib provided statistically-significant survival benefits compared with placebo in patients with advanced HCC. Because survival is influenced by second- and beyond-line therapies, reviews of other cancers demonstrate the requirement for post-progression survival (PPS) analysis [5,6,7].

However, sorafenib only induces modest tumor shrinkage. Cytotoxic therapy is most often discontinued or changed when radiological progressive disease (rPD) occurs; however, the optimal termination point for targeted therapy can be difficult to determine according to rPD [3,4,8]. So-called clinical PD (cPD) does not correspond to rPD during sorafenib therapy for HCC, because we experienced a few patients who continued sorafenib after diagnosis of rPD retaining disease control over a long period in clinical practice. The Response Evaluation Criteria in Solid Tumors (RECIST) was originally developed to assess responses to cytotoxic agents and may not be appropriate for targeted agents [9,10].

This study analyzed prognostic factors for PPS using patient parameters at rPD. We then attempted to develop an indicator for judging cPD as an adjunct to RECIST using these prognostic factors for patients with advanced HCC administered sorafenib as first-line therapy.

## 2. Experimental Section

The Saga Liver Cancer Study Group (SALC) comprises tertiary-care hospitals in Saga, Japan, with specialists in liver cancer treatment. A retrospective analysis of all patients with HCC treated with sorafenib in Saga Prefecture was performed using the unified database system of the SALC. The patients received 400 mg of sorafenib twice daily; however, initial dose reduction considering each patient’s condition was allowed. All other aspects of sorafenib therapy, including dose adjustment or interruption, treatment schedule, supportive therapies and post-rPD therapies, were determined by a physician. The institutional review board or ethics committee of each institution approved the SALC protocol.

All patients had histologically- or radiologically-confirmed HCC that was diagnosed as advanced, ineligible for resection or locoregional treatment or refractory to chemoembolization. Patients’ conditions when sorafenib treatment was initiated were defined according to the Eastern Cooperative Oncology Group performance status (ECOG PS) as scores ranging from 0 to 2, Child–Pugh scores ≤8 and adequate hematologic and liver function. Adequate hematologic functions were defined as follows: hemoglobin concentration ≥8.5 g/dL, neutrophil count >1500/µL and platelet count >75,000/µL. Adequate liver functions were defined as alanine aminotransferase and aspartate aminotransferase levels lower by a factor of 5 than the normal upper limit and total bilirubin level <3.0 mg/dL. Patients requiring hemodialysis were not included. Patients were also considered ineligible if they received concomitant systemic therapy, including any targeted agents. All patients provided written informed consent before receiving sorafenib therapy.

The physician determined the first rPD. Radiologic evaluations were conducted every 4–8 weeks using enhanced computed tomography or magnetic resonance imaging, according to RECIST Version 1.1 [11]. Time to progression (TTP) was calculated from the date of initial sorafenib administration to the date of first rPD, or was censored at the last follow-up, or at the time of death without rPD. PPS was the primary endpoint and was calculated from the date of first rPD until the date of death or last follow-up.

For this analysis, patients with first rPD were classified into three groups according to post-rPD management as follows: patients who continued to receive sorafenib beyond rPD for at least 1 month (Group A), patients who received other second-line therapies after rPD (Group B) and patients who received best supportive care (BSC) (Group C). Groups A and B were integrated into one post-rPD therapy group (Group A + B) and compared with Group C. Post-rPD management was decided by the physician in accordance with the patient’s condition. 

Distributions of variables among the groups were compared using the χ^2^ test for categorical data and the Mann–Whitney *U* test for continuous data. TTP and PPS were estimated using the Kaplan–Meier method, and the curves were compared using the log-rank test. Cox proportional hazards models were used to identify factors associated with PPS. The models were also used to devise the scoring system for judging the first rPD. Univariate analyses were performed to assess potential factors related to PPS. If the factors attained a significance level of *p* < 0.01, a multivariate proportional hazard model for these factors was constructed to estimate each coefficient (β) and hazards ratio. Scores were defined according to the weighted sum of those factors, with weights defined simply as the estimated coefficients. Cutoff value, sensitivity and specificity for PPS were assessed using the time-dependent receiver-operating characteristic (ROC) curve [12] and area under ROC (AUROC) curves. ROC curves were plotted with 1-specificity and sensitivity measured along the horizontal and vertical axes, respectively. All statistical analyses were performed using R Version 3.0.1 (The R Foundation for Statistical Computing, Vienna, Austria).

## 3. Results and Discussion

### 3.1. Results

#### 3.1.1. Patient Characteristics and Outcomes

Sorafenib was administered to 101 patients from July, 2008–April, 2012, at five SALC institutions. Seventy-six patients experienced their first rPD, and the remaining 25 patients were either alive without rPD (*n* = 17) or discontinued sorafenib caused by adverse events before rPD (*n* = 8). Among the patients documented with rPD, 26 were in Group A, 29 in Group B and 21 in Group C. Group A (beyond rPD) and Group B (second-line therapy) were integrated into one group (Group A + B), and the patient characteristics for the A + B and C groups at the first rPD are listed in Table 1. At the first rPD, ECOG PS, Child–Pugh class and the level of α-fetoprotein (AFP) of Group C were significantly worse than those of Group A + B. Dose reduction or interruption of sorafenib were required for 45 patients (81.8%) in Group A + B and 12 patients (57.1%) in Group C (*p* = 0.027). The median TTP was significantly longer in Group A + B than in Group C (2.5 *vs.* 1.8 months, respectively; *p* = 0.012). Similarly, median PPS was also significantly longer in Group A + B than in Group C (9.5 *vs.* 2.1 months, respectively; *p* < 0.001).

**Table 1:** Patient characteristics.

Variable	Group A + B *n* = 55	Group C *n* = 21	*p*
At initiation of sorafenib treatment			
Sex, n (%)			
Male	42 (76)	17 (81)	0.670
Female	13 (24)	4 (19)	
Therapy before sorafenib, n (%)			
Surgical resection	23 (42)	8 (38)	0.769
Locoregional ablation	29 (53)	12 (57)	0.732
Transarterial chemoembolization	45 (82)	15 (71)	0.324
Median number of therapies before sorafenib	4	5	0.420
At the first rPD			
Median age, years	74	77	0.406
ECOG PS, n (%)			
0, 1	51 (93)	12 (57)	<0.001
≥2	3 (5)	9 (43)	
Unknown	1 (2)	0	
Cause of liver disease, n (%)			
HCV	29 (53)	17 (80)	0.074
HBV	9 (16)	2 (10)	
Others	17 (31)	2 (10)	
Child–Pugh class, n (%)			
A	35 (64)	5 (24)	<0.001
B	19 (34)	11 (52)	
C	0	5 (24)	
Unknown	1 (2)	0	
BCLC stage, n (%)			
B	21 (38)	5 (24)	0.241
C	34 (62)	16 (76)	
Incidence of severe AEs, n (%)	30 (55)	10 (48)	0.591
History of treatment interruption, n (%)	30 (55)	10 (48)	0.591
Best antitumor response ^†^, n (%)			
Partial response	2 (4)	1 (5)	0.424
Stable disease	22 (40)	5 (24)	
Progression disease	31 (56)	15 (71)	
Median AFP, ng/mL	77	2,506	0.032
AFP >1000 ng/mL, n (%)	14 (25)	11 (52)	0.023

^†^ According to the Response Evaluation Criteria in Solid Tumors (RECIST) Version 1.1 until the first rPD. Abbreviations: AEs, adverse events; AFP, α-fetoprotein; BCLC, Barcelona Clinic Liver Cancer; ECOG PS, Eastern Cooperative Oncology Group performance status; HBV, hepatitis B virus; HCV, hepatitis C virus; rPD, radiological progressive disease.

#### 3.1.2. Prognostic Factors for PPS

The following variables at the first rPD based on previous reports [3,4,13,14,15,16,17,18,19,20,21,22] and clinical experiences were selected for analysis as follows: age, gender, ECOG PS, Child–Pugh class, Barcelona Clinic Liver Cancer stage, best antitumor response until first rPD using RECIST Version 1.1, tumor shrinkage, contrast enhancement disappeared lesion, adverse events caused by sorafenib, course of treatment and AFP level. In univariate analyses, ECOG PS ≥2 points (*p* < 0.001), Child-Pugh B (*p* < 0.001), Child-Pugh C (*p* < 0.001), PD as the best antitumor response (*p* = 0.004) and AFP level >1000 ng/mL (*p* = 0.002) were statistically-significant factors for PPS. These factors were selected as independent factors affecting PPS in multivariate analysis (Table 2).

**Table 2:** Cox proportional hazard model analysis of post-progression survival (PPS).

Variable	Univariate	Multivariate		
	*p*	β	HR (95% CI)	*p*
Age > 70 years	0.090			
Male	0.646			
ECOG PS ≥ 2	<0.001	0.943	2.568 (1.317–5.006)	0.006
Child-Pugh Class A			1	
B	<0.001	0.846	2.329 (1.173–4.624)	0.016
C	<0.001	3.200	24.525 (5.860–102.635)	<0.001
BCLC Stage C	0.414			
PD as the best antitumor response	0.004	1.162	3.195 (1.625–6.279)	0.001
Absence of tumor shrinkage	0.918			
Absence of contrast enhancement disappeared lesion	0.398			
History of AEs ≥ Grade 3	0.468			
History of interrupted treatment	0.457			
AFP > 1000 ng/mL	0.002	0.912	2.490 (1.327–4.673)	0.005

Abbreviations: AEs, adverse events; AFP, α-fetoprotein; BCLC, Barcelona Clinic Liver Cancer; CI, confidence interval; ECOG PS, Eastern Cooperative Oncology Group performance status; post-progression survival; HR, hazard ratio; PD, progression disease; PPS, post-progression survival.

#### 3.1.3. Post-rPD Therapy

**Figure 1:**
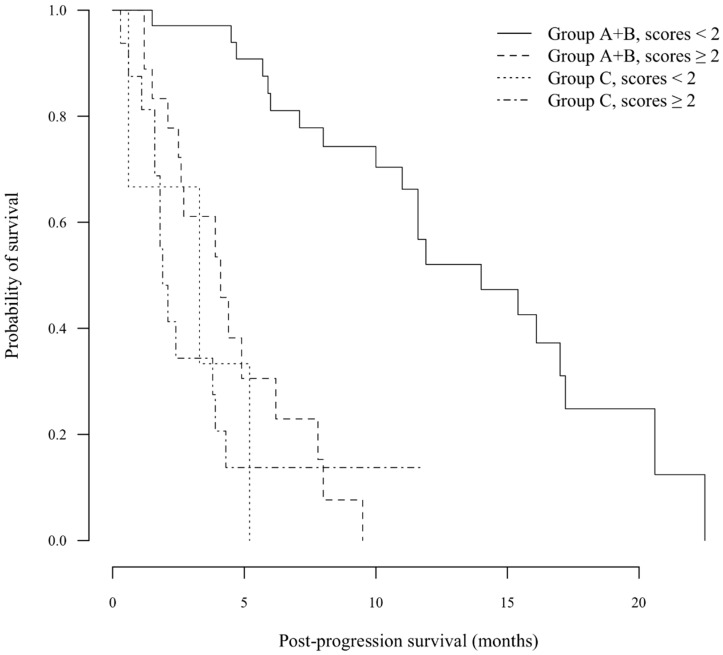
Kaplan–Meier analysis of post-progression survival (PPS). In patients with scores ≥2 points, no statistically-significant difference in PPS was observed between the A + B and C groups (median 4.1 *vs.* 1.9 months, respectively; *p* = 0.220). In contrast, the PPS of Group A + B was significantly longer than that of Group C in patients with scores <2 points (median 14.0 *vs.* 3.3 months, respectively; *p* < 0.001).

To determine an indicator for judging cPD at the first rPD, the scoring system for PPS was designed using the factors described above (Table 3). The median score was significantly lower for Group A + B than Group C (one *vs.* three points, respectively; *p* < 0.001). The AUROC was 0.856 in time-dependent ROC for PPS according to Group C. The cutoff value for the scoring system of two points yielded a theoretical sensitivity of 71.5% and a theoretical specificity of 87.5%. When scores were limited to ≥2 points, no statistically-significant difference in PPS was observed between Groups A + B and C (median 4.1 *vs.* 1.9 months, respectively; *p* = 0.220). In contrast, the PPS of Group A + B was significantly longer than that of Group C in patients with scores of zero or one (median 14.0 *vs.* 3.3 months, respectively; *p* < 0.001) (Figure 1), although there were only three patients in Group C with scores of zero or one.

**Table 3:** Scoring system for PPS.

Variables at the first rPD	Score		
	0	1	2
Child-Pugh class	A	B	C
ECOG PS	0, 1	≥2	
Best response ^†^	CR/PR/SD	PD	
AFP	<1000 ng/mL	≥1000 ng/mL	

^†^ According to the RECIST Version 1.1 until the first rPD. Abbreviations: AFP, α-fetoprotein; CR, complete response; ECOG PS, Eastern Cooperative Oncology Group performance status; PPS, post-progression survival; PR, partial response; RECIST, Response Evaluation Criteria in Solid Tumors; rPD, radiological progressive disease; SD, stable disease.

#### 3.1.4. Beyond rPD

When patient characteristics were compared between Groups A and B, there were no statistically-significant differences with respect to the variables at baseline or first rPD as described above in comparisons between the A + B and C groups. With reference to treatment duration of sorafenib after first rPD in Group A, median time to sorafenib withdrawal was 2.7 months. Post-rPD therapies of Group B were as follows: transarterial chemoembolization (*n* = 14), systemic chemotherapy with fluoropyrimidines (*n* = 7), hepatic arterial infusion chemotherapy (*n* = 4), other targeted therapy (*n* = 3) and radiofrequency ablation (*n* = 1).

The median PPS was 8.0 months for Group A and 10.0 months for Group B (*p* = 0.310). Sixteen and 18 patients in Group A and B, respectively, scored between zero and one. Their prognoses were better, and median PPS was comparable between the two groups (both median 11.6 months; *p* = 0.537). In contrast, patients with scores ≥2 points, PPS was poorer compared with those with scores of zero or one (median 3.9 *vs.* 11.6 months, respectively; *p* < 0.001).

### 3.2. Discussion

The present study identifies prognostic factors for PPS in patients treated with sorafenib, and we propose a numerical indicator for cPD. Prognoses for patients with scores ≥2 were very poor; PPS did not vary significantly between Groups A + B and C. Therefore, these patients could be judged as cPD, and BSC would be recommended for treating these patients after the first rPD.

Although tumor response has been used in clinical research as a surrogate indicator of survival, the survival benefit of sorafenib for advanced HCC does not correlate with decreased tumor size [3,4]. Therefore, distinguishing disease progression using conventional response criteria can be difficult, and vigorous research has been conducted to identify other surrogate indicators or biomarkers to assess the effects of sorafenib on patients with HCC. For example, AFP levels and AFP response correlate with survival outcomes [3,16,17]. Similarly, ECOG PS and Child-Pugh class correlated with the prognosis of patients with advanced HCC who were treated with sorafenib [3,19,20]. The modified RECIST (mRECIST) is widely used to assess the effect of treatment on patients with HCC [22]. The data of the present study were also evaluated with mRECIST, and the same results were obtained.

PPS was comparable between patients in Groups A and B who continued sorafenib beyond rPD, as well as for patients treated using other therapies after rPD. For patients with metastatic colorectal cancer treated with first-line therapy that included bevacizumab, continuing bevacizumab beyond progressive disease increased survival, showing the efficacy of continuous inhibition of angiogenesis for cancer therapy [23,24,25]. Moreover, tumor rebound occurs after discontinuing anti-angiogenic therapy [26,27,28]. Second-line therapy after sorafenib failure is not well established for patients with advanced HCC, although several randomized trials are in progress to define salvage therapy [29,30,31]. A randomized phase 2 study showed that increasing the dose of sorafenib beyond progressive disease resulted in a trend, although not statistically significant, toward improved progression-free and overall survival, compared with BSC administered to patients with progressive disease previously treated with sorafenib [32]. Our present results demonstrate that continuation of sorafenib beyond rPD may provide a therapeutic option for patients with scores of zero or one until promising second-line regimens become available. However, we were unable to identify patients more likely to respond to continued sorafenib treatment beyond rPD. The number of patients in our study was limited. Moreover, it is possible that the lower score in Group A + B might be reflected by a selection bias because post-rPD management relied on physician’s decision in this retrospective study. To overcome these limitations, further studies are required to define the role of post-rPD therapy. We are conducting a prospective study to validate the efficacy of sorafenib therapy beyond rPD and the reliability of the score.

## 4. Conclusions

Child-Pugh class, ECOG PS, the best antitumor response during treatment and AFP levels were identified as independent factors affecting PPS. We propose a useful scoring system as an adjunct to RECIST for predicting rPD in patients with advanced HCC. Because prognoses for patients with scores ≥2 at first rPD were very poor, BSC may be recommended in these patients with cPD until promising second-line therapy becomes available.
